# Update on congenital glaucoma

**DOI:** 10.4103/0301-4738.73683

**Published:** 2011-01

**Authors:** Anil K Mandal, Debasis Chakrabarti

**Affiliations:** VST Centre for Glaucoma Care, L V Prasad Eye Institute, L V Prasad Marg, Banjara Hills, Hyderabad - 500 034, India; 1 Suryodaya Eye Centre, The Calcutta Medical Research Institute, 7/2, Diamond Harbour Road, Kolkata-700027, India

**Keywords:** Congenital glaucoma, goniotomy, trabeculotomy, trabeculectomy and combined trabeculotomy–trabeculectomy

## Abstract

Congenital glaucoma is a global problem and poses a diagnostic and therapeutic challenge to the ophthalmologist. A detailed evaluation under general anesthesia is advisable to establish the diagnosis and plan for management. Medical therapy has a limited role and surgery remains the primary therapeutic modality. While goniotomy or trabeculotomy ab externo is valuable in the management of congenital glaucoma, primary combined trabeculotomy–trabeculectomy offers the best hope of success in advanced cases. Trabeculectomy with antifibrotic agent and glaucoma drainage devices has a role in the management of refractory cases, and cyclodestructive procedures should be reserved for patients where these procedures have failed. Early diagnosis, prompt therapeutic intervention and proper refractive correction are keys to success. Management of residual vision and visual rehabilitation should be an integral part of the management of children with low vision and lifelong follow-up is a must.

Once upon a time, the pediatric glaucomas were considered mostly untreatable and synonymous with inevitably profound visual loss. However, with better understanding of the disease at the genetic level, newer technological tools assisting in diagnosis, newer intraocular pressure (IOP)-lowering medications and refinement of surgical techniques, we are better equipped today to restore or preserve the sight of children afflicted with glaucomas. This paper provides an overview and the current view on congenital glaucoma.

## Historical Perspective

Hippocrates (460–377 BC), Celsus (1st century AD) and Galen (130–201 AD) had recognized congenital enlargement of the eye, but did not relate the condition to elevated IOP. Berger (1744) mentioned increased IOP, but it was in 1869 that von Muralt established the classical type of buphthalmos within the family of glaucoma. However, both von Muralt and von Graefe considered that the condition was caused by a primary intraocular inflammation. It was in the late 1800s and early 1900s that precise anatomical dissections were carried out and the etiopathology pointed toward various malformations of the structures of the angle of the anterior chamber (AC). Such studies were conducted by von Hippel (1897), Parsons (1904), Siegrist (1905), Gros (1897), Reis (1905–1911), Seefelder (1906–1920), and others.

In the early 1900s, the congenital glaucomas seemed untreatable and Anderson commented, “the future of patients with hydrophthalmia is dark” and “one seeks in vain for a best operation in the treatment of hydrophthalmia”.[1] The poor prognosis of infantile glaucoma changed dramatically in 1938[2] with the introduction of goniotomy (Greek: *gonio* = angle; *tomein* = cut) by Otto Barkan, who revived Italian surgeon de Vincentis’ operation (1892), which “incised the angle of the iris in glaucoma”. Otto Barkan modified de Vincentis’ operation by using a specially designed glass contact lens to visualize angle structures while using a knife to create an internal cleft in the trabecular tissue. Barring instrumental and microscopic development, the operation has essentially remained unchanged.

In 1949, Barkan described a persistent fetal membrane overlying the trabecular meshwork. This was confirmed by Worst (1966), who termed it Barkan’s membrane. However, pathological studies by Anderson, Hansson, Maul, Maumenee, and others could not find the existence of any such membrane by light or electron microscopy.

For many years, goniotomy remained the classic operation for congenital glaucoma, till a new technique called “trabeculectomy ab externo” was described simultaneously and independently by Burian and Smith in 1960. In March 1960, without the aid of an operating microscope, the first external trabeculectomy was performed by Burian on a young girl with Marfan syndrome and glaucoma. In the same year, Redmond Smith developed an operation which he called “nylon filament trabeculotomy”. This involved cannulating Schlemm’s canal with a nylon suture at one site, threading the suture circumferentially, withdrawing it at another site, and pulling it tight like a bow-string. The surgical technique of trabeculotomy ab externo was subsequently modified by Harms (1969), Dannheim (1971) and McPherson (1973).

## Terminology

### General terms

1. Buphthalmos (Greek: *bous* = ox; *ophthalmos* = eye) refers to the marked enlargement that can occur as a result of any type of glaucoma present since infancy.
2. Hydrophthalmos (Greek: *hydro* = water; *ophthalmos* = eye) refers to the high fluid content present with marked enlargement of the eye, seen in any glaucoma present since infancy.

Both are mere descriptive terms and do not imply etiology or appropriate therapy, hence should not be used diagnostically.

### Relating to age of onset

1. Congenital glaucoma: The glaucoma exists at birth, and usually before birth.
2. Infantile glaucoma: Occurs from birth until 3 years of life.
3. Juvenile glaucoma: Occurs after the age of 3 to teenage years.

### Relating to developmental pattern

Developmental glaucoma: Glaucoma associated with developmental anomalies of the eye present at birth.

- Primary developmental glaucoma: Resulting from maldevelopment of the aqueous outflow system.
- Secondary developmental glaucoma: Resulting from damage to the aqueous outflow system due to maldevelopment of some other portion of the eye, e.g., angle closure due to pupillary block in a small eye, or an eye with microspherophakia or dislocated lens; or as a forward shift of the lens-iris diaphragm in persistent hyperplastic primary vitreous or retinopathy of prematurity.

### Relating to structural maldevelopment

1. Goniodysgenesis, 2. trabeculodysgenesis, 3. irido-dysgenesis and 4. corneodysgenesis refer to the maldevelopment of the irido-corneal angle, trabecular meshwork, iris and cornea, respectively. These may present either singly or in some combination. Isolated trabeculodysgenesis is the hallmark of primary developmental glaucoma.

We will mainly focus on primary congenital glaucoma (PCG) in our discussion.

#### Classification

Several classification systems are in vogue, such as the Shaffer–Weis classification (1970), DeLuise–Anderson classification (1983) and the anatomical classification by Hoskins–Shaffer–Hetherington (1984). The last system has prognostic implications (isolated trabeculodysgenesis, e.g., responds more favorably to surgical intervention compared to trabeculodysgenesis associated with iris or corneal abnormalities).

- **Isolated trabeculodysgenesis**
  1. Flat iris insertion
    - Anterior insertion
    - Posterior insertion
    - Mixed insertion
  2. Concave (wrap-around) iris insertion
  3. Unclassified
- **Iridotrabeculodysgenesis**
  1. Anterior stromal defects
    - Hypoplasia
    - Hyperplasia
  2. Anomalous iris vessels
    - Persistence of tunica vasculosa lentis
    - Anomalous superficial vessels
  3. Structural anomalies
    - Holes
    - Colobomata
    - Aniridia
- **Corneotrabeculodysgenesis**
  1. Peripheral, e.g., Axenfeld’s anomaly
  2. Midperipheral, e.g., Rieger’s anomaly
  3. Central e.g., Peter’s anomaly, anterior staphyloma, AC cleavage syndrome, or posterior corneal ulcer of von Hippel
  4. Corneal size, e.g., microcornea or macrocornea

### Epidemiology

PCG is a rare eye disorder which accounts for 0.01–0.04% of total blindness. The disease is usually manifested at birth or early childhood (before 3 years of age). The incidence of PCG is different in different populations. In western developed countries, the incidence is approximately 1 in 10,000 births.[3] The incidence of PCG is increased when “founder effect” or a high rate of consanguinity are found in a population. The “founder effect” is a gene mutation observed in high frequency in a specific population due to the presence of that gene mutation in a single ancestor or small number of ancestors. The incidence is 1 in 1250 in the Slovakian Roms (Gypsies),[4] 1 in 2500 in the Middle East,[5] and 1 in 3300 in Andhra Pradesh, India.[6] In Andhra Pradesh, the disease accounts for 4.2% of all childhood blindness.[6]

The majority of patients (about 60%) are diagnosed by the age of 6 months, and 80% are diagnosed within the first year of life. A slight predominance of males is common (about 65%), and involvement is usually bilateral (about 70%).

Most cases of PCG occur sporadically. Patients with a familial pattern usually show a recessive pattern with incomplete or variable penetrance and possibly multifactorial inheritance. An autosomal dominant and pseudo-dominant mode of inheritance has also been reported. The disease is familial in 10–40% of cases with variable penetrance (40–100%).[7,8]

#### Genetic studies

Loci of recessively inherited PCG (gene GLC3) have been identified by genetic linkage analysis [Table 1].[9,10]

**Table 1:** Known genetic loci for primary congenital glaucoma

Locus	Location	Inheritance	Mutated gene (MIM number)
GLC3A	2p21	AR	*CYP1B1* (601771)
GLC3B	1p36	AR	Unknown

AR: Autosomal recessive, MIM: Mendelian inheritance in man

The majority of congenital glaucoma map to GLC3A locus on chromosome 2 (2p21). Families linked to these loci display severe phenotypes with autosomal recessive inheritance pattern. Some types of juvenile onset glaucoma with autosomal dominant inheritance pattern have been mapped to chromosome 1q23-q25 (*TIGR/MYOC* gene).

Mutations in the *CYP1B1* gene (encoding the cytochrome P450 enzyme) in the GLC3A locus are associated with the PCG phenotype. This is the predominant genetic pattern of PCG in the Middle East (Turkey and Saudi Arabia). It has been reported that 87% of familial and 27% of sporadic cases are due to mutations in this gene.[11]

Digenic inheritance is an inheritance mechanism resulting from the interaction of two non-homologous genes. Digenic inheritance in glaucoma has been shown recently in two instances: in early-onset glaucoma in humans and also in the mouse. *CYB1B1* and *MYOC* mutations were identified in early-onset glaucoma in humans, whereas mutations in the *CYP1B1* and *FOXC1* were detected in the mouse with early-onset glaucoma.[12,13] Because angle structures are mainly derived from neural crest cells, it is possible that defects in genes expressed in neural crest cells could also contribute to PCG.

*CYP1B1* mutations have also been noted in Indian patients. Reddy and co-workers screened 146 PCG patients from 138 pedigrees and reported six distinct *CYP1B1* mutations from 45 patients. These include four novel mutations [ins 376A or Ter@223(frameshift), P193L, E229K, and R390C] and two known mutations (G61E and R368H). Of the mutations identified, R368H was the predominant mutation causing PCG in India. This allele was found in a very low proportion of patients from the Middle East and Brazil, but 16.2% of screened patients in India had this mutation.[14]

Of the mutations studied, frameshift and R390C homozygous mutations were associated with very severe phenotypes and very poor prognoses. This approach may help gene therapy and counsel the afflicted family regarding the likelihood of progression of the disorder.

Genetic studies have also been conducted on a few other glaucomas. In Axenfeld-Rieger anomaly, the gene has been mapped to chromosome 6p25 region.[15] A few mutations in a forkhead/winged-helix transcription factor gene *FOXC1* have been implicated in the pathogenesis of this disorder.

In aniridia, the most common inheritance pattern is autosomal dominant. The genetic locus for aniridia has been established as the *PAX6* gene, which is located on the 11th chromosome (11p13).[16]

## Primary Congenital Glaucoma

PCG refers to a specific form of developmental glaucoma characterized by an isolated maldevelopment of the trabecular meshwork (isolated trabeculodysgenesis) not associated with other developmental ocular anomalies or ocular disease that can raise the IOP. Also called primary infantile glaucoma, it is the most common form of developmental glaucoma. The condition is typically bilateral, but 25–30% of the cases may be unilateral.

**Figure 1:**
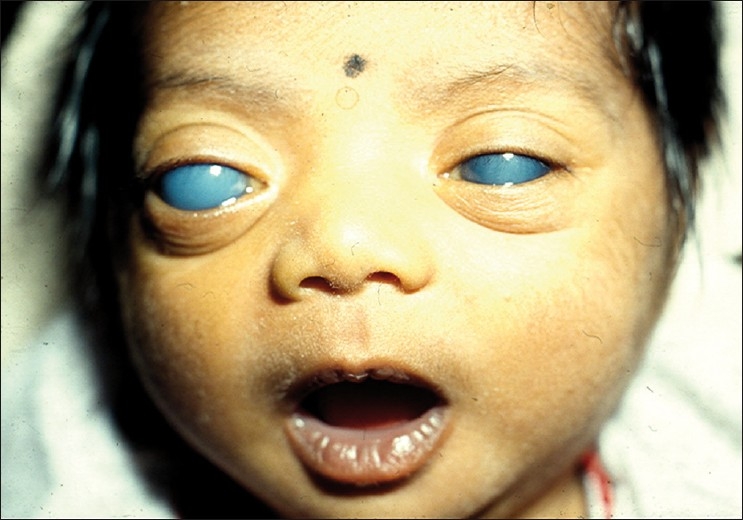
Primary congenital glaucoma

Most western textbooks describe a classic triad of symptoms comprising epiphora, photophobia and blepharospasm (attributable to IOP-induced corneal epithelial edema). However, one study conducted in a tertiary institution shows that large eyeball size and hazy eyes (from corneal edema) may be the more common presenting features in the Indian subcontinent[17] [Fig. 1]. Occasionally, the child may also present with a red eye, mimicking conjunctivitis.

A thorough clinical evaluation is mandatory. Useful information can be obtained in the Out Patient Department (OPD) itself be capturing the child’s attention with a slowly blinking flashlight or a gentle jingle of keys.[18] Vision is assessed by age-appropriate methods. Whenever the posterior segment cannot be visualized, it is advisable to perform an ultrasonography (USG) B-scan to rule out any posterior segment pathology.

The mainstay of evaluation remains the examination under anesthesia (EUA). We prefer to examine under the operating microscope. For a brief examination, only intravenous and mask anesthesia is often sufficient. For more prolonged examination and surgery, an endotracheal tube is necessary. The points to be noted and documented during EUA are the following:

1. Refraction, using streak retinoscope
2. *Corneal findings:* diameter, clarity, Haab’s striae
  - A corneal diameter greater than 12 mm in the first year of life is highly suggestive of PCG.[19] Corneal enlargement from PCG predominantly occurs before the age of 3 years, but the sclera may be deformable until approximately 10 years of age. Both the horizontal and vertical meridians are measured separately, but the vertical meridian may be difficult to measure accurately due to encroachment of the sclera at the superior limbus. Most commonly, calipers are used for the measurements.
  - Corneal edema in PCG is initially simple epithelial edema due to elevated IOP. Subsequently, there is permanent stromal edema. Untreated, the edema progresses to stromal scarring and irregular corneal astigmatism.
  - The increased IOP stretches the corneal endothelium and Descemet’s membrane, resulting in breaks in these layers, first described by Haab in 1863. These striae are typically horizontal and linear when they occur centrally in the cornea, but parallel or curvilinear to the limbus when they occur peripherally.
3. *IOP:* The type of anesthesia and the type of tonometer are important. All anesthetics alter the IOP, seemingly in relation to the level of anesthesia and as a direct function of their effect on cardiovascular tone. A rapid lowering of IOP occurs particularly with halothane, while transient elevation can occur with cyclopropane or succinylcholine. In recent years, halothane has been largely replaced by sevoflurane because of its rapid onset of action, faster recovery time and lower incidence of reported side effects. However, sevoflurane shares the IOP-lowering effect of halothane and it has been suggested that IOP should be checked immediately after intubation to avoid falsely low recordings. Anesthetic drugs that achieve only light anesthesia and those that induce deeper anesthesia only slowly, such as diethyl ether, cyclopropane or ketamine, allow the IOP to be measured somewhere between the artificially elevated IOP of “excitement” stage of anesthesia and the artificially lowered pressure of deep anesthesia observed with halothane. The possible effect of ketamine on IOP has been controversial. Some studies report that ketamine elevates IOP, whereas others suggest that the impact on IOP is comparatively modest. Regarding the type of tonometer, Perkins hand-held applanation tonometer or electronic (Tonopen) tonometer is commonly employed. Our preference is the Perkins tonometer. The normal IOP in an infant is slightly lower than in an adult, but 21 mm Hg remains a useful upper limit.[18]
4. Gonioscopy: The Koeppe 14–16 mm lens with a hand-held slit-lamp or Barkan light and hand-held binocular microscope provides a good view of the angle. In the normal newborn eye, the iris usually inserts posterior to the scleral spur. In PCG, the iris commonly inserts anteriorly directly into the trabecular meshwork. This iris insertion is most commonly flat, although a concave insertion may be rarely seen. Although the angle is usually avascular, loops of vessels from the major arterial circle may be seen above the iris (“Loch Ness Monster phenomenon”). In addition, the peripheral iris may be covered by a fine, fluffy tissue (“Lister’s morning mist”).
5. *Ophthalmoscopy:* A direct ophthalmoscope or a Koeppe contact lens can be used for this purpose. The infant glaucomatous cup usually has a configuration different from adult glaucomas. Although it can be oval, it is more commonly round, steep-walled and central, surrounded by a uniform pink rim. The cup tends to enlarge circumferentially with glaucomatous progression, which probably results from stretching of the scleral canal. Although this pattern of cupping can develop early and rapidly in infants with glaucoma, striking reversal of cupping may result with IOP reduction after successful glaucoma surgery. By contrast, both adults and children (especially older ones) with advanced glaucoma suffer irreversible thinning/notching of neuro-retinal rims.

*Interpretation of examination findings:* In most cases, after completion of EUA, the findings of corneal enlargement, optic nerve head changes and buphthalmos are so typical of PCG that there is little doubt about the diagnosis and the need for surgery. If the IOP is normal and the other findings are present, one can assume that the IOP is artifactually lowered under anesthesia, and still secure the diagnosis and proceed with surgery. If ocular enlargement and optic nerve cupping are not typical or are absent, it is appropriate to postpone the diagnosis and treatment until a repeat EUA is performed after 3–4 weeks to confirm any progression.

## Management

### Overview of management

Medical therapy usually provides a supportive role to reduce the IOP temporarily, to clear the cornea, and to facilitate surgical intervention. Laser therapy has a limited role in developmental glaucomas. The most effective and definitive form of treatment of most developmental glaucomas is surgical. Primary surgical treatment is usually with goniotomy or trabeculotomy, although combined trabeculotomy with trabeculectomy may be useful in certain populations with a high risk of failure of goniotomy or trabeculotomy. Refractory pediatric glaucomas may be managed by trabeculectomy with anti-fibrosis drugs, glaucoma drainage implants and cyclodestructive procedures.

### Medical therapy

As discussed, medical therapy usually plays a supportive role. In one study, in 161 eyes with congenital glaucoma, medical therapy alone reduced the IOP to less than 21 mm Hg in 12% of eyes in the short term and 10% of eyes in the long term.[20]

1. *Beta-blockers:* Beta-blockers have been extensively studied. In 34 patients with childhood glaucoma, timolol was added to other medical therapy, causing a definite improvement in 29%, a modest or equivocal improvement in 32% and no improvement in 39%.[21] In 38 eyes treated with timolol as adjunctive therapy, 37% of eyes were controlled at 22 mm Hg or less.[22] In 100 eyes with childhood glaucoma treated with Timolol, 31% experienced a reduction in IOP.[23] Timolol in 0.25% and 0.5% solutions may be used cautiously in young glaucoma patients. The drug should be used with extreme caution in neonates due to the possibility of apnea and other systemic side effects. Cardiac abnormalities and bronchial asthma should be specifically excluded before its use. Use of 0.25%, rather than 0.5%, is recommended in children in order to reduce its side effects; however, the 0.25% formulation is not widely available. Hence, 0.5% timolol can be used with punctal occlusion.
2. *Carbonic anhydrase inhibitors (CAIs):* Systemic CAIs would be expected to have similar side effects in children as compared to adults. In addition, growth suppression in children has been associated with oral acetazolamide therapy, and infants may experience a severe metabolic acidosis. Oral administration of acetazolamide suspension at a dosage of 10 (range 5–15) mg/kg/day given in divided doses (three times daily) is safe and well tolerated by children, lowers IOP and may reduce corneal edema as a prelude to surgery. Topical versus oral CAI therapy has been evaluated for pediatric glaucoma in a crossover design study.[24] The mean IOP was reduced by 36 and 27% compared with baseline, after treatment with oral acetazolamide and topical dorzolamide, respectively. All eyes showed an increase in IOP when switched from acetazolamide to dorzolamide, with a mean increase of 3.7 mm Hg. Although not as effective as acetazolamide in this group of patients, topical dorzolamide caused a significant reduction of IOP and was well tolerated. At present, topical CAIs are more commonly prescribed compared with systemic CAIs. For older children, the fixed combination of dorzolamide with timolol may simplify medical regimen, reducing the number of drops instilled per day.
3. *Prostaglandin analogues:* In one study using latanoprost in 31 eyes with a variety of glaucoma diagnoses, 6 (19%) of the treated eyes responded with a decrease of IOP averaging 8.5 mm Hg (34% reduction), whereas the majority of eyes were non-responders. Responders were more likely to have juvenile-onset open-angle glaucoma and to be older than non-responders.[25] However, some studies on children show a significant ocular hypotensive effect with latanoprost. Parents should be advised about the possibility of local side effects, including iris pigmentation, eyelash elongation and hyperemia.
4. *Alpha-2 agonists:* In 30 patients with a mean age of 10 years, brimonidine treatment was associated with a mean reduction of IOP by 7%.[26] Two young children (2 and 4 years of age) were transiently unarousable, and five other children experienced extreme fatigue.[26] In another study involving 23 patients with a mean age of 8 years, 18% had systemic adverse effects that necessitated stopping the drug.[27] Because of these central nervous system mediated side effects, brimonidine should be used with caution in pediatric patients, and only be used in older children.
5. *Other drugs:* In pediatric patients, the use of pilocarpine is limited. Osmotic drugs like mannitol may be administered to reduce the IOP before surgery in patients with developmental glaucomas with IOPs that are high even with standard medical therapy.

### Surgical treatment

#### Introduction

Early surgical intervention is of prime importance in the management of patients with developmental glaucoma. In some areas of the world, such as United States, patients may have only mild or moderate corneal edema at referral for treatment. These patients may be candidates for goniotomy, which has a high success rate in the western population. In other areas of the world, such as India or the Middle East, nearly all patients present with clouding and goniotomy is technically impossible. In these areas, external trabeculotomy is the initial procedure of choice. When initial trabeculotomy has a poor success rate, trabeculotomy may be combined with trabeculectomy. Another important factor is that although most patients have symptoms suggestive of congenital glaucoma at birth or within 6 months of birth, they often present late due to various non-medical factors. In such advanced cases, we prefer to perform ab externo combined trabeculotomy with trabeculectomy, which offers the best hope of success.[18]

#### Goniotomy

The objective of goniotomy is to remove the obstructing tissue that causes resistance to aqueous outflow, thereby restoring the access of aqueous to Schlemm’s canal and maintaining the physiologic direction of outflow. The most common gonioscopy lens used is the Swan-Jacob lens. Barraquer knife, Worst knife, Swan spade or even a long needle can be used as a goniotomy knife. We prefer to use the operating microscope as the viewing system. Under general anesthesia (GA), the locking fixation forceps is placed by the assistant on the vertical recti for a goniotomy performed nasally or temporally. A paracentesis is performed with a sharp blade, or the goniotomy knife itself is used to enter the AC. The knife enters the AC through peripheral clear cornea, approximately 1 mm inside the corneoscleral junction, at the previously selected site. Once in the AC, the knife is guarded parallel to the iris, away from the pupil, toward the trabecular meshwork. The tip of the knife is engaged slightly anterior to the middle of the trabecular meshwork. With the operating microscope, the tip of the knife can be seen to indent the trabecular meshwork before it is cut by circumferential movement of the knife. The tip of the knife is kept in a somewhat superficial position, cutting at the same depth along the incision. As the incision proceeds, a white line develops behind the blade, the iris falls posteriorly, and the angle deepens. By rotation of the globe by the assistant, approximately 4“6 clock-hours of meshwork can be excised. The reported results of goniotomy surgery show a success rate of 80% in infantile glaucoma.[28] It appears that goniotomy is most successful in patients whose glaucoma is recognized early and treated between 1^st^ month and one year of age. The use of an endoscope for goniotomy surgery is a relatively new concept and only few animal studies and case reports have been published in the literature.[29]

#### Trabeculotomy ab externo

Trabeculotomy ab externo has a number of advantages over the alternative operation of goniotomy. It can be done even if the cornea is hazy, can accomplish rupture of the inner wall of the Schlemm’s canal and trabecular meshwork with anatomical precision, does not require the introduction of sharp instruments across the AC and can be done with standard microsurgical technique without the need of having to adapt with the view of goniotomy lens. Advocates of trabeculotomy state that the success of this operation depends only on the type of angle anomaly and is not dependent on the severity of the glaucoma, the size of the cornea or the presence of corneal edema, which has been reported to influence the success of goniotomy. Considering congenital glaucoma of all grades of severity, goniotomy controls the IOP in about 64–77% of eyes, while trabeculotomy controls IOP in over 90% of eyes. However, there are no prospective, controlled trials to compare the success rate of these procedures in the same study.

The procedure essentially involves creation of a fornix-based or limbus-based conjunctival flap, creation of a triangular or rectangular superficial scleral flap, deroofing the Schlemm’s canal by incising at the junction of the bluish-gray zone anteriorly and the white scleral zone posteriorly, then introducing the metal trabeculotome on each side and entering into the AC after rupturing the inner wall of the Schlemm’s canal, followed by suturing the scleral flap with 10-0 nylon and the conjunctival flap with 8-0 vicryl.[18] Several studies have reported that as the initial procedure, trabeculotomy enjoys a higher success rate than goniotomy, but some studies showed that they are equally effective. A newer trabeculotomy technique with a prolene suture passed 360° through the Schlemm’s canal has been described.[30]

#### Primary trabeculectomy

Trabeculectomy is a procedure that most ophthalmologists are familiar with and is technically easier than goniotomy or trabeculotomy. However, many authors do not consider it as a first-line procedure in congenital glaucoma in view of a higher incidence of complications and lower success rate. Nevertheless, several reports have documented successful results following primary trabeculotomy for congenital glaucoma, which are comparable to goniotomy or trabeculotomy.[31]

#### Trabeculectomy with mitomycin-C

When one or more angle procedures (and medications) have failed to achieve IOP control in refractory primary infantile glaucoma cases, filtering surgery is often undertaken. Although most glaucoma filtration surgery in children was standardly performed using a limbus-based conjunctival flap, many surgeons now advocate fornix-based flaps in both infants and children. The use of intraoperative mitomycin-C has somewhat enhanced the success of this procedure, although not without significant and, as yet, unquantifiable risk. Mandal and associates reported success of 66% at 30 months in a series of 38 eyes, most of which had refractory PCG.[32] Other authors have reported varying rates of success over relatively short follow-up times, using mitomycin-C doses ranging from 0.2 to 0.5 mg/ml, applied for from 2 to 5 minutes; bleb-related infections have been reported in most of these series.[33–36]

#### Combined trabeculotomy with trabeculectomy

**Figure 2:**
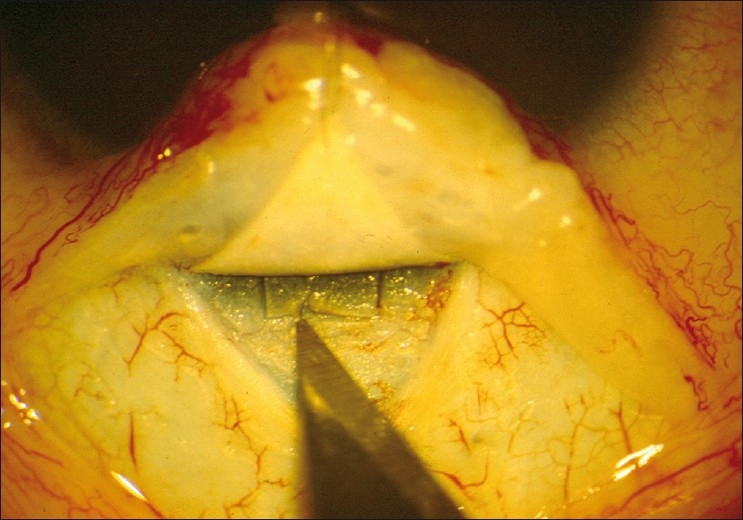
Anatomy of the limbal region identified and Schlemm’s canal is explored

**Figure 3:**
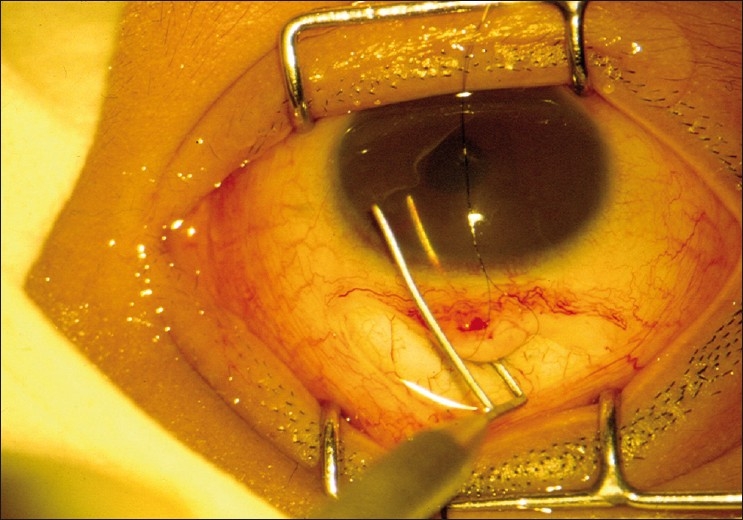
Trabeculotomy probe rotated into AC from the left side

**Figure 4:**
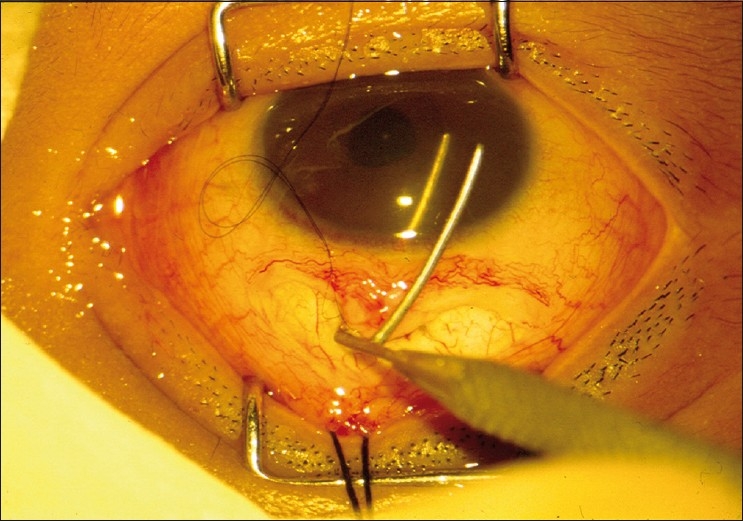
Trabeculotomy probe rotated into AC from the right side

**Figure 5:**
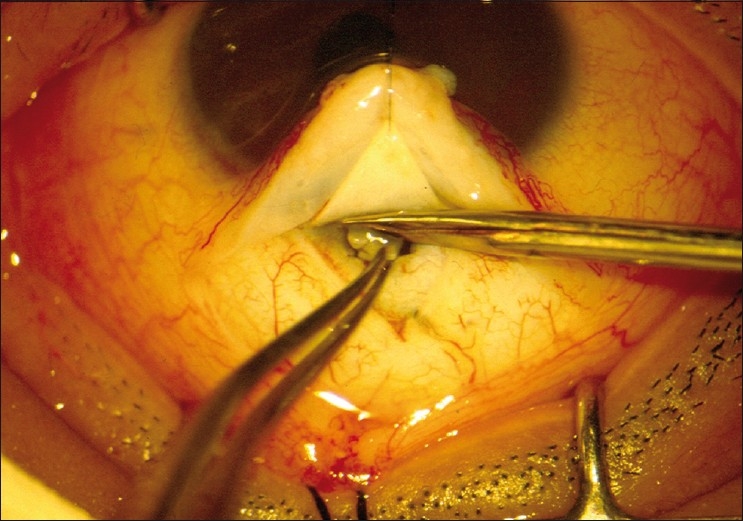
The deeper trabecular block is removed

**Figure 6:**
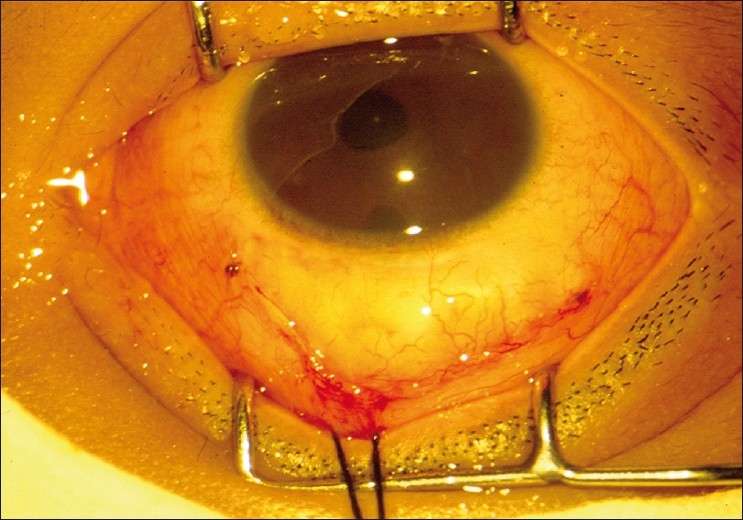
Closure of the conjunctival incision with 8-0 vicryl

After performing a trabeculotomy, a block of sclera is removed from the deep scleral flap by Vannas scissors or punch, as in a routine trabeculectomy [Figs. 2–6].

**Figure 7:**
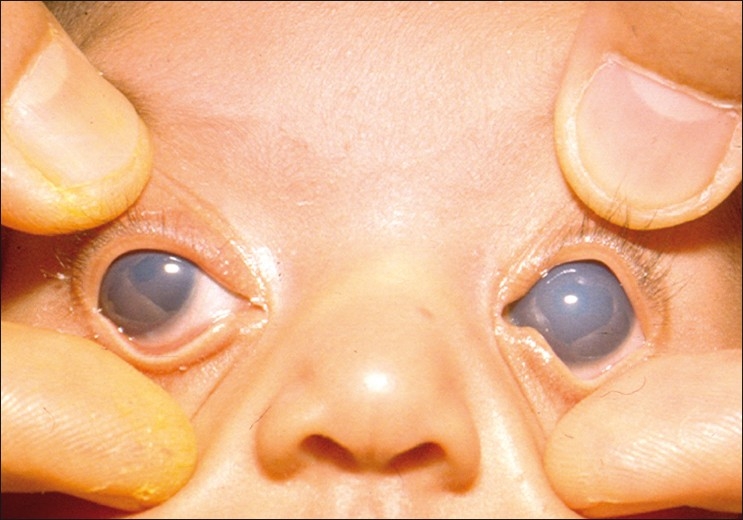
Primary congenital glaucoma operated at the age of 1 week

**Figure 8:**
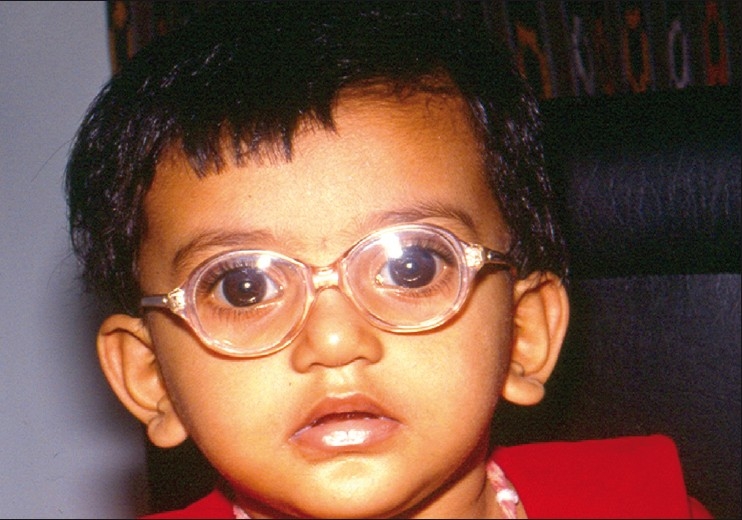
Three years postoperative appearance of the same child with normal vision

**Figure 9:**
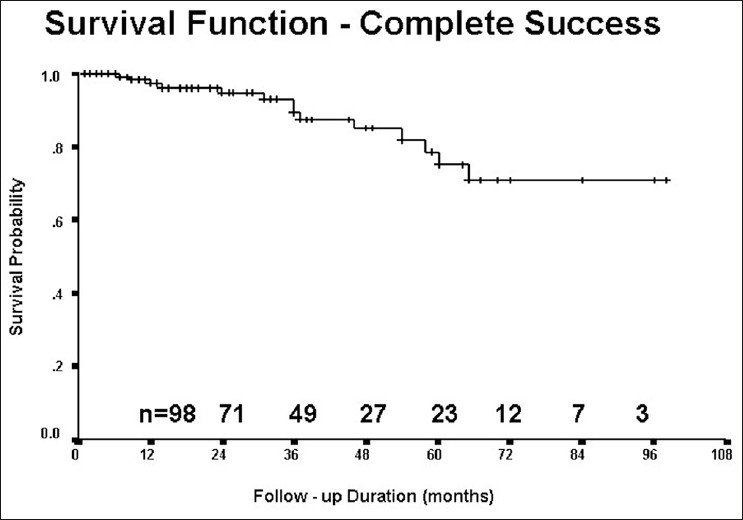
Long-term success after combined trabeculotomy– trabeculectomy

Whether combined trabeculotomy–trabeculectomy is superior to trabeculotomy alone is debatable. Biedner and Rothkoff found no difference between the two in a small series of seven patients.[37] Dietlin *et al*. investigated the outcome of trabeculotomy, trabeculectomy and a combined trabeculotomy–trabeculectomy; although initially the combined procedure seemed to have a favorable outcome, after 2 years this difference was not statistically significant.[38] Elder compared primary trabeculectomy with combined trabeculotomy–trabeculectomy and found the combined procedure to be superior.[39] Mullaney *et al*.[40] and Al-Hazmi *et al*.[41] used mitomycin-C in primary combined trabeculotomy–trabeculectomy and reported a higher success rate. Mandal *et al*.,[42] from India, reported similar success rates but did not use mitomycin-C in primary surgery. Mandal *et al*.[43] also reported long-term outcome of 299 eyes of 157 patients who underwent the combined surgery; the success rate of 63.1% was maintained until 8 years of follow-up [Figs. 7–9]. Combined trabeculotomy–trabeculectomy is safe and effective in advanced primary developmental glaucoma with corneal diameter 14 mm or more.[44] Mandal *et al*.[45] reported 624 eyes of 360 consecutive patients who underwent primary combined trabeculotomy–trabeculectomy for primary developmental glaucoma between January 1990 and June 2004. They concluded that prolonged IOP control can be achieved in patients with primary developmental glaucoma and 42% of the patients gained normal visual acuity.

Whatever surgery is performed, the responsibility of the surgeon does not end with the surgery and care must be taken to manage amblyopia for optimum visual rehabilitation of the child. Myopia and astigmatism are often present in infants with PCG. In unilateral cases, the affected eye is usually more myopic. Astigmatism often results when Haab’s striae cross the visual axis. The amblyopia can be attributed to stimulus deprivation (due to corneal haziness) and anisometropia. Following successful surgery, glasses should be prescribed after refraction; and amblyopia should be treated by patching – more important for unilateral cases.

## Management of Refractory Pediatric Glaucomas

When the IOP is not controlled after the first surgery, the surgical options are filtration surgery with anti-fibrosis drugs, glaucoma drainage implants or cyclodestructive procedures.

**Figure 10:**
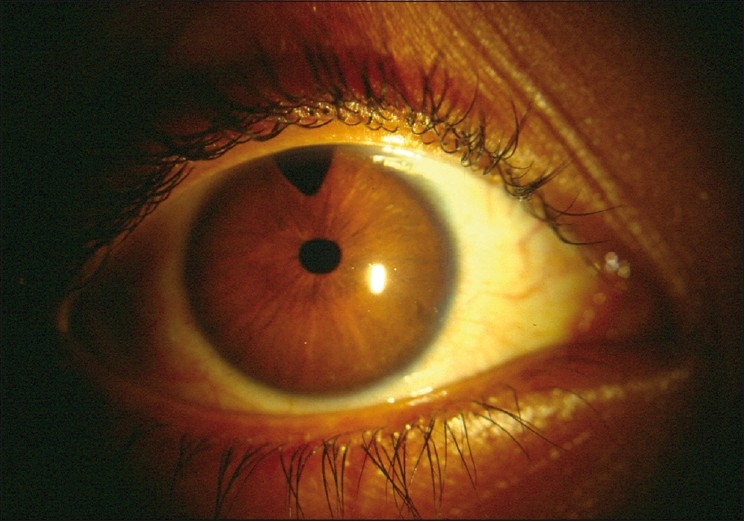
Anterior segment photograph after failed primary surgery

**Figure 11:**
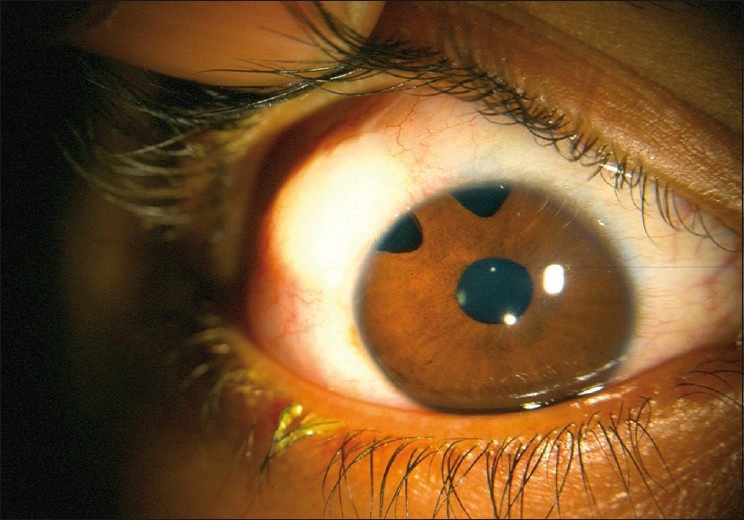
Anterior segment photograph showing bleb appearance after mitomycin-C augmented trabeculectomy

The authors do not prefer trabeculectomy with mitomycin-C as the primary surgery because of the potential complications of mitomycin-C and also because of the reported higher success rates of alternative procedures like combined trabeculotomy–trabeculectomy. However, several studies have reported good success rates of trabeculectomy with mitomycin-C in children, the success rate ranging from 48 to 95%, depending on the patient’s age, definition of success, duration of follow-up and other factors. We consider filtering surgery with antimetabolites, a useful option in refractory congenital glaucoma with previously suboptimal primary surgical results [Figs. 10 and 11]. The reported range of concentration varies from 0.2 to 0.4 mg/ml, with an exposure time of 2–4 minutes. We prefer 0.4 mg/ml for 2 minutes.

Glaucoma drainage implants may be characterized as open-tube (non-restrictive) devices such as the Molteno and Baerveldt implants, or valved (flow-restrictive) devices such as Krupin implant or Ahmed glaucoma valve. The flow-restrictive devices are intended to reduce the incidence of complications associated with hypotony during the immediate postoperative period. Both the Baerveldt and Ahmed (S3 and FP8) implants, the most commonly used varieties, come in smaller sizes, which may be more fitting for the pediatric eye. Children may be particularly prone to extrusion and exposure of the implant. The reported success rate of non-valved implants is 68–86% and that of valved implants is 40–91%.[46] The Ahmed valve comes equipped with a flow-restricted device designed to decrease the surface area than the Baerveldt, which may adversely affect ultimate IOP lowering and predispose to a more significant hypertensive phase. Implantation may be easier with the Ahmed, particularly in a small orbit. Due to the flow-restrictive device, the tube may be allowed to function immediately, another advantage of the valved device. Reported complications of implants include hypotony with shallow AC and choroidal detachments, tube-cornea touch, obstructed tube, exposed tube or plate, endophthalmitis and retinal detachment.

The most commonly performed cyclodestructive procedures are cyclophotocoagulation (transscleral Nd:YAG, transscleral diode and endoscopic diode) and cyclocryotherapy. When available, the former is preferable because of less postoperative inflammation and possibly less incidence of phthisis. Cyclophotocoagulation can be performed with a variety of lasers, including the Nd:YAG, 810 nm diode, and krypton laser. The laser is delivered by a specially designed contact probe applied near the limbus. Success rates range from 28 to 79%.[47] An intraocular technique employing an endoscope with endolaser has been described for more precise delivery of laser energy to the target tissue. In general, cyclodestructive procedures are often unpredictable as to the extent of IOP control, have limited success rates, often require re-treatment, and may be associated with vision-threatening complications. Some clinicians advocate these procedures early in the treatment regimen, while most reserve them until other treatments have not succeeded. Supplemental submaximal or full treatment with cyclophotocoagulation may be useful if the IOP is uncontrolled despite glaucoma drainage implants or other surgical treatments.

For refractory glaucomas, the authors prefer trabeculectomy with mitomycin-C as the initial approach; if results are unsatisfactory, we proceed to Ahmed glaucoma valve. However, the exact order of treatment is dependent on the individual surgeon preference at this time.

## Management of Residual Vision in Pediatric Glaucoma

Unfortunately, even with the best treatment in the best centers, many children with congenital glaucomas end up with “low vision”. Visual rehabilitation and low vision aids can help these children lead a normal or near-normal life. After proper assessment, telescopes (hand-held or spectacle-mounted) may be prescribed to improve distant vision while hand or pocket magnifiers (2× to 3×) may be prescribed to improve near vision. Structured training programs in the use of these devices should be planned and discussed with the child and parents.

## Conclusion

Once considered virtually untreatable, pediatric glaucomas have now become reasonably manageable in most cases, thanks to scientific advances. Let us pray that ongoing research in this field will assist to further help the preservation or restoration of vision of children suffering from glaucomas.
